# Cellular miR-150-5p may have a crucial role to play in the biology of SARS-CoV-2 infection by regulating *nsp10* gene

**DOI:** 10.1080/15476286.2021.2010959

**Published:** 2021-12-14

**Authors:** Shaw M. Akula, Paul Bolin, Paul P. Cook

**Affiliations:** Department of Microbiology & Immunology (S.m. Akula), Department of Internal Medicine (P. Bolin, P.P.Cook), Brody School of Medicine at East Carolina University, Greenville, NC, USA

**Keywords:** COVID-19, SARS-CoV-2, miR-150-5p, nsp10, plasma, circulating biomarkers

## Abstract

The role for circulating miRNAs as biomarkers of the COVID-19 disease remains uncertain. We analysed the circulating miRNA profile in twelve COVID-19 patients with moderate-severe disease. This analysis was conducted by performing next generation sequencing (NGS) followed by *real-time* polymerase chain reaction (RT-qPCR). Compared with healthy controls, we detected significant changes in the circulating miRNA profile of COVID-19 patients. The miRNAs that were significantly altered in all the COVID-19 patients were miR-150-5p, miR-375, miR-122-5p, miR-494-3p, miR-3197, miR-4690-5p, miR-1915-3p, and miR-3652. Infection assays performed using miRNA mimics in HEK-293 T cells determined miR-150-5p to have a crucial role in SARS-CoV-2 infection and this was based on the following data: (i) miR-150-5p mimic lowered *in vitro* SARS-CoV-2 infection; (ii) miR-150-5p inhibitor reversed the effects of miR-150-5p mimic on SARS-CoV-2 infection of cells; and (iii) a novel miRNA recognition element (MRE) was identified in the coding strand of SARS-CoV-2 *nsp10*, the expression of which could be inhibited by miR-150-5p mimic. Our findings identified crucial miRNA footprints in COVID-19 patients with moderate-severe disease. A combination of co-transfection and Western blotting experiments also determined the ability of miR-150-5p to inhibit SARS-CoV-2 infection via directly interacting with MRE in the coding strand of *nsp10*. Our investigation showed that a sharp decline in the miR-150-5p plasma levels in COVID-19 patients may support enhanced SARS-CoV-2 infection. Furthermore, this study provides insight into one possible mechanism by which COVID-19-induced changes to miR-150-5p levels may promote SARS-CoV-2 infection via modulating *nsp10* expression.

## Introduction

A worldwide public health emergency has been caused by a novel coronavirus termed SARS-CoV-2, the causative agent of coronavirus disease 2019 (COVID-19). The pandemic has affected millions and shows no signs of abating. Multiple treatment options have been evaluated, including chloroquine, lopinavir/ritonavir, azithromycin, remdesivir, monoclonal antibodies to IL-6, and others [1–3]. In a short time period, we have expanded our knowledge base on SARS-CoV-2 (the pathogen), host–virus interactions including the receptor molecules that empower the virus to enter cells, key genes that are modulated during the course of COVID-19, and therapies to manage the disease *per se*. Vaccines developed based on different platforms have already been approved for use, and vaccination programmesare in full swing. However, knowledge on the roles of microRNAs (miRNAs) in the biology of COVID-19 is still at its infancy.

miRNAs are small non-coding RNAs (ncRNA) [4–10], nucleotides in length, derived from hairpin-shaped precursor molecules encoded by the genomes of animals, plants, and viruses [11]. miRNAs control gene expression and regulate a wide array of biological processes by targeting messenger RNAs (mRNA) and inducing translational repression or RNA degradation [12]. Among mammals, miRNA coding sequences are estimated to account for about 1% of the genome, yet greater than 60% of protein coding genes are regulated by miRNAs [13]. To date, there are limited reports published on the role of miRNAs in the biology of COVID-19. Most of these reports on miRNAs are based on computational predictions [14], *in vitro*-based assays [15], focused on a particular protein such as ACE-2 related miRNAs [16], differential miRNA expression in blood derived from COVID-19 patients [17–19], or reviews [20]. Determining the host response to SARS-CoV-2 infection in terms of changes to miRNA expression profiles is critical to understanding the biology of COVID-19.

The focus of this study was to determine the key circulatory miRNAs that are significantly altered in COVID-19 patients with moderate–severe disease using the whole transcriptome assay. Over-expression of cellular miR-150-5p that was down-regulated in COVID-19 patients could inhibit SARS-CoV-2 infection of target cells, *in vitro*. Further analysis determined a possible interaction between cellular miR-150-5p and a unique miRNA recognition element (MRE) on the SARS-CoV-2 encoded non-structural protein 10 (*nsp10*) gene. The *nsp10* gene product has a crucial role to play in viral replication and in evading host immune response [21]. The nsp10 protein binds to nsp14 and nsp16 to activate their 3ʹ to 5ʹ exoribonuclease (ExoN) and methylation activity, respectively [22]; both of which aid in translation efficiency, immune evasion, and SARS-CoV-2 replication. The ramifications of these findings are discussed below.

## Materials and Methods

### SARS-CoV-2

All the work pertaining to the use of SARS-CoV-2 were performed in BSL-3 laboratory. SARS-CoV-2 (isolate USA-WA1/2020) purchased from bei RESOURCES (Manassas, VA) was propagated in Vero cells as per standard procedures [23]. The virus yield was titrated in Vero cells, and theTCID_50_ was calculated using the Reed and Muench formula. The following research was approved by the Office of Prospective Health/Biological Safety for the use of biohazardous agent (SARS-CoV-2) and the registration number is 20–01 (title: Host response to COVID-19 infection in Eastern North Carolina).

### Cells

Human embryonic kidney cells (HEK-293 T), Vero, and human lung adenocarcinoma-derived Calu-3 cells were propagated in Dulbecco modified Eagle medium (DMEM) (Invitrogen, Carlsbad, CA) containing 10% charcoal-stripped foetal bovine serum, L-glutamine, and antibiotics as per earlier standard protocols [24].

### Plasmid

The full-length *nsp10* gene (13,025–13442bp) with the wild-type MRE (13168bp – 13189bp; 5ʹ-cactggtactggtcaggcaata-3ʹ) cloned in pCMV3 (*nsp10/*pCMV) was used in this study. We also generated *nsp10* gene possessing a MRE mutant (*nsp10M/*pCMV) (5ʹ-caAAAAAActggtcaggcaata-3ʹ) as per our earlier studies [25] and the manufacturer’s recommendations using the QuickChange II Site-Directed Mutagenesis kit (Agilent, Santa Clara, CA) [4]. Both these plasmids were used in transfection assays as per standard protocols using FuGene HD reagent as per our laboratory procedures [5].

### Antibodies

Polyclonal SARS-CoV-2 *nsp10* antibodies (GeneTex, Irvine, CA; Cat No. GTX135733) and mouse anti-actin antibodies (Clone AC-74; Sigma-Aldridge) were used in this study.

### Human participants

This was a single-centre study. The patient plasma samples were obtained from the Department of Pathology, Vidant Medical Center, Greenville, NC. These specimens were from COVID-19 positive patients with moderate-severe disease. The patient details and the demographics are provided in Tables 1 and 2, respectively. The inclusion criteria for the COVID-19 participants were, (i) age ≥18 years; (ii) hospitalized ≤48 hours; (iii) confirmed SARS-CoV-2 by PCR; and (iv) moderate-severe pneumonia as assessed by radiographic imaging and oxygen requirements. All subjects were requiring supplemental oxygen at the time of the blood sampling. Plasma from healthy volunteers were used as controls. The healthy volunteers were apparently healthy people who did not exhibit symptoms of COVID-19 or any other form of respiratory distress in the past 14 days. None of them were vaccinated for COVID-19 as it was not available at that period of time. We age-matched the healthy controls by choosing healthy volunteers aged between 30–66 years of age.

**Table 1.** Patient details

Patient ID	Age (years)	Co-Morbidities	CRP (mg/l)*	Serum 25-OH-vitamin D (ng/ml)*	ALC (cells/µl)*
A	60	HTN, asthma, DM, obesity	219.5	39.4	330
B	38	DM	48.4	48.7	830
C	48	Renal transplant, HTN, DM	ND	7.0	160
D	45	HTN, DM, prostate cancer	199.1	35.9	860
E	56	ESRD, HTN, DM	127.1	7.5	500
F	39	HTN, DM, obesity	112.5	53.9	910
G	45	DM, obesity	22.7	11.2	1800
H	33	HTN, COPD, paraplegia	254.5	40.2	1680
I	49	HTN, CHF, cirrhosis	154.2	14.7	1420
J	66	HTN, DM, obesity	302.1	27.3	1500
K	40	Obesity	125	7.0	600
L	66	HTN, DM, obesity	ND	8.0	200

* Values obtained at the time of admission.

CRP = C-reactive protein

HTN = Hypertension

DM = Diabetes Mellitus

ESRD = End Stage Renal Disease

COPD = Chronic Obstructive Pulmonary Disease

CHF = Congestive Heart Failure

**Table 2.** Sample information

Parameters	COVID-19 patients (n = 12)	Healthy volunteers (n = 8)
**Age, y, mean (SD)**	47.8 (9.8)	46 (7.3)
**Sex, n (%)**		
**Male**	6 (50%)	3 (37.5%)
**Female**	6 (50%)	5 (62.5%)
**Sample collected**	Blood (plasma)	Blood (plasma)

### Study design and approval

COVID-19 positive human plasma samples were obtained from the department of Pathology, East Carolina University. Control plasma samples were obtained from healthy participants with informed written consent before inclusion in the study in accordance with Declaration of Helsinki principles. All protocols were approved by the University and Medical Center IRB (UMCIRB) review board. The approved UMCIRB study number is UMCIRB 20–001604.

### Whole transcriptome assay

We determined the circulating miRNA profile in the procured human plasma specimens by performing the HTG EdgeSeq miRNA Whole Transcriptome Assay (miRNA WTA) (HTG Molecular Diagnostics, Inc. Tucson, AZ). The HTG EdgeSeq miRNA WTA enables us to measure the expression of 2,083 human miRNA transcripts using next-generation sequencing (NGS). The plasma samples (20 µl) were incubated with 20 µl plasma lysis buffer plus 4 µl of proteinase-K (miRNA lysis buffer kit; HTG Molecular Diagnostics) with constant shaking at 50֯C for 3 h to denature them. The miRNA expression profile was determined as per standardized protocols [6]. Briefly, the samples were loaded into an HTG Edgeseq Processor. After the automated preparation process, libraries were prepared with TruSeq Small RNA Prep kit (Illumina). Single-end reads of 51 bp in length were sequenced using an Illumina GAIIx instrument. To quantify the expression levels, trimmed reads were mapped to the genome and duplications were removed. Finally, the annotation from miRBase v20 was used to designate reference mapped reads to mature miRNAs.

### RT-qPCR

RNA was extracted from the plasma specimens as per standard laboratory procedures using miRNeasy Serum/Plasma Advanced kit (Qiagen, Germantown, MD). The RNA concentrations were measured with a NanoDrop ND-2000 spectrophotometer (Thermo Fisher Scientific, Waltham, MA, USA). Only the RNA samples with 260/280 ratios of 1.8 to 2.0 were used in the study. The isolated plasma miRNA (20 µl vol) was incubated with 1 U (1 µl vol) of heparinase I (St. Louis, MO) [7] and 20 U (1 µl vol) RiboLock RNase inhibitor (Thermo Fisher Scientific) at room temperature for 30 minutes. This is a critical step to minimize the inhibitory heparin from all the patient-derived plasma.

Heparinase I-treated RNA was reverse transcribed in a 25 µl reaction volume using the All-in-oneTM miRNA RT-qPCR detection kit (GeneCopoeia, Rockville, MD, USA). Briefly, the cDNA was synthesized in a 25 μl reaction mix containing 5 μl of 5x PAP/RT buffer, 2.5 U/μl poly A polymerase, and 1 µl RTase mix. The reaction was performed at 37°C for 60 min and terminated at 85°C for 5 min. cDNA that was produced in the RT reaction was directly used as the template for the PCR reaction in a Bio-Rad iQ5 Multicolour Real-Time PCR (Bio-Rad, Hercules, CA). In this system, MS2 RNA was used as an external reference for the quality of the extracted miRNAs, and RNU6B, RNU44, RNU48, and RNU49 were used for normalization. The expression levels of miRNAs were measured employing RT-qPCR with the SYBR green detection and specific forward primer for the mature miRNA sequence and the universal adaptor reverse primer (GeneCopoeia, USA). The specific forward primer to amplify different miRNAs was used (**Supplementary Table S1**). Data were excluded from further analyses, if Cq-values exceeded 35 and/or the amplification score was below 1.5.

### Cytotoxicity assay

The LDH assay was performed using the CytoTox 96 non-radioactive kit (Promega, Madison, WI) as per earlier studies [8]. Target cells were treated with different concentrations of miR-36 mimic and inhibitor at 37°C in a V-bottom 96-well plate. After a 24 h incubation, the cells were analysed for the expression of LDH, as an indicator of cell death. The LDH assay was performed using the CytoTox 96 non-radioactive kit (Promega) as per earlier studies [8].

### Infection assay, and effect of miRNA mimics on SARS-CoV-2 infection

HEK-293 T cells were used to monitor the effects of miRNA mimics on SARS-CoV-2 infection. Briefly, target cells were transfected with different concentrations of various miR-mimics, or scramble control (miR-SC); miRNA inhibitor to miR-150-5p (Inh-150-5p) and non-specific inhibitor (Inh-NS) (Sigma-Aldridge) using FuGene HD reagent as per our laboratory procedures [5]. At 24 h post transfection, these cells were infected with 1 multiplicity of infection (MOI) of SARS-CoV-2 for 2 h at 37֯C. Unadsorbed virus were removed by washing cells twice with DMEM and further incubating with infection medium at 37֯C. On 3^rd^ day post infection (PI), the supernatant was collected, and the virus in terms of 50% tissue culture infective dose (TCID_50_) was determined by performing standard titration in Vero cells. The Reed and Muench formula was used to calculate TCID_50_ [23]. The virus concentration in the supernatant collected was also detected by RT-qPCR using the 2019-nCoV RUO kit as per the protocols outlined by the manufacturer (Integrated DNA technologies, Coralville, IA).

### Binding assay

The effect of miR-150-5p mimic and inhibitor on SARS-CoV-2 binding to HEK-293 T cells was assessed by PCR detecting the cell-bound SARS-CoV-2 [9]. Briefly, untransfected cells or cells transfected with 50 nM concentration of miR-mimic or 25 nM concentration of inhibitor were infected with 10 MOI of SARS-CoV-2 at +4֯C. After 60 min of incubation with virus, cells were washed three times with PBS to remove the unbound virus. Cells were pelleted, and total RNA including those representing the cell-bound SARS-CoV-2 was isolated using RNeasy kit (Qiagen, Valencia, CA) and subjected to RT-qPCR analysis monitoring nucleocapsid N gene (accession number: NC_045512) using the SARS-CoV-2 (2019-nCoV) CDC qPCR Probe Assay (Integrated DNA Technologies). The amplicon size is 72bp and the annealing temperature is 55֯C. All primer pairs used in this study were predicted by BLAST to be specific under the default stringency. Incubating SARS-CoV-2 with 100 µg/ml of heparin (Sigma-Aldridge) for 1 h at 37֯C was used as known positive control.

### Dual-luciferase reporter assay

The 3ʹ-UTR (with wild-type binding sites for miR-3197, and miR-3652, respectively) of GPX2 (accession number NM_002083: amplicon size – 250 bp; annealing temperature – 55֯C), and AGO3 (accession number: NM_024852: amplicon size – 250 bp; annealing temperature – 55֯C), respectively, was PCR amplified and then cloned into pMIR-report luciferase vector (Thermo Fisher Scientific). HEK-293 T cells were plated onto 6-well plates. At 24 h post-plating, HEK-293 T cells were co-transfected with GPX2 (or AGO3) 3′-UTR luciferase reporter plasmid and miR-3197 mimic (or miR-3652 mimic), and 40ng pRL-TK Renilla luciferase reporter plasmid using FuGene HD (Promega). Appropriate miR-inhibitors were used as controls in this study. At 48 h post transfection, the Renilla luciferase activity was measured using the dual-luciferase reporter assay system (Promega) as per manufacturer’s recommendations.

### Western blotting

All the buffers used in this project were made with water that was endotoxin and pyrogen free. Western blotting was conducted as per earlier studies using the following primary antibodies to SARS-CoV-2 nsp10 and human β-actin. The bands were scanned, and the band intensities were assessed using the ImageQuaNT software program(Molecular Dynamics).

### Statistics

The data satisfied normality (K-S test, p > 0.1); they were used untransformed unless stated otherwise. Student *t* test or One-way analysis of variance (ANOVA) was appropriately performed to determine the statistical significance. ANOVA was performed using IBM SPSS v26 (Cary, NC) to determine significant differences between the treatment and control groups, followed by Tukey HSD post-hoc test for multiple comparisons. The *P value* was established at the 0.05 level.

## Results

### Differential expression of miRNAs in COVID-19 patients as determined by EdgeSeq technology

**Figure 1.**
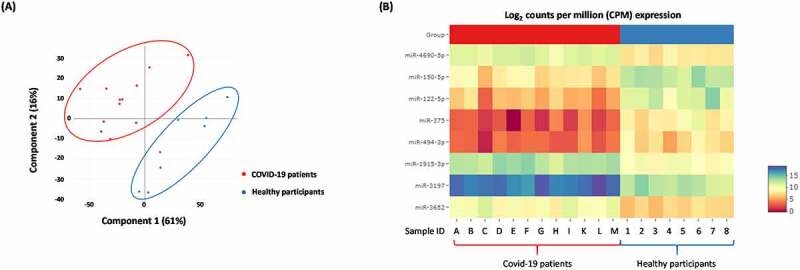
**Differentially expressed 1018 miRNAs in COVID-19 patients with moderate-severe disease compared to healthy participants**. (A) Principal component analysis (PCA) plot showing transcriptome differences between miRNA expression in COVID-19 patients compared to healthy participants. Each dot represents one of the total number of samples (12 samples from COVID-19 patients and 8 samples from healthy participants) used in the sequencing-based screening. (B) Heat map showing the expression of specific miRNAs in COVID-19 patients compared to healthy participants. The red and blue colours indicate up-regulation (log2 [2.5]) and down-regulation (log2 [−2.5]), respectively as shown in the colour bar.

**Figure 2.**
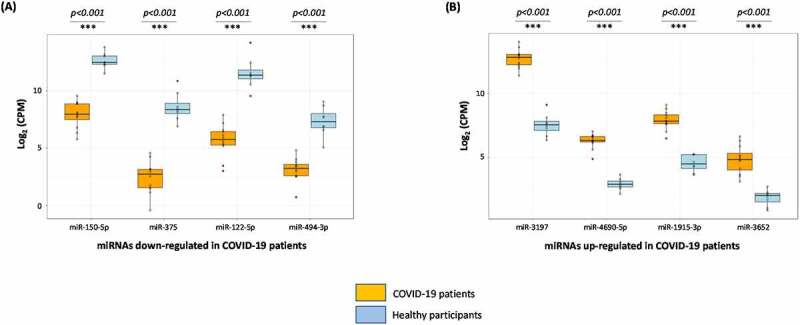
**Boxplots of the 8 miRNAs differentially expressed in COVID-19 patients with moderate-severe illness**. Screening of 2083 human miRNA transcripts using extraction-free HTG EdgeSeq system determined 8 differentially expressed circulating miRNAs in the plasma of COVID-19 patients when compared to healthy participants. miRNAs down-regulated (A) or up-regulated (B) are grouped and analysed separately in box plots. Student *t* test was performed to compare miRNA expression levels between the two (COVID-19 patients vs healthy control) groups. Two-tailed *P* value of 0.05 or less was considered statistically significant. The level of statistical significance is also marked where *** denotes *P < 0.001.*

**Figure 3.**
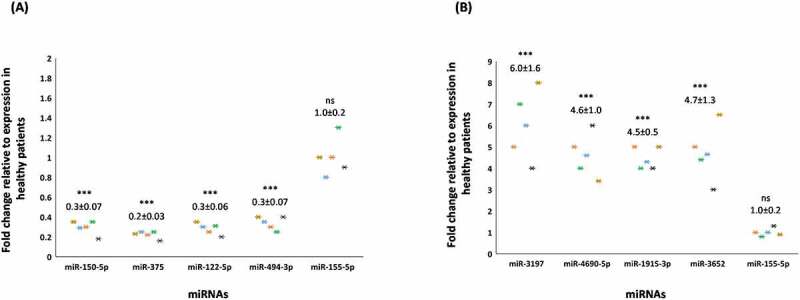
**Validation of NGS-determined miRNA candidates by RT-qPCR**. Fold changes in the average expression of the down-regulated (A) and up-regulated (B) miRNAs in COVID-19 patients is depicted in the plots. The fold change is relative to the average expression of respective miRNAs in healthy participants and that is considered to be 1-fold. Expression of miR-155-5p was used as a control in this study. We used miR-155-5p as a control because the circulatory levels of it in the COVID-19 patients was comparable to what was observed in healthy participants. The average ± s.d. of five individual experiments is listed above the data points. One-way analysis of variance (ANOVA) was performed using IBM SPSS v26 (Cary, NC) to determine significant differences between the groups, followed by Tukey HSD post-hoc test for multiple comparisons. The level of statistical significance is also marked with three (***) if *P < 0.001*; ns – not significant.

The clinical details of all patients are summarized in Table 1. By using the extraction-free HTG EdgeSeq system and bioinformatical analyses, the expression of 2083 circulating miRNAs were analysed in the plasma derived from COVID-19 patients when compared to apparently healthy participants. Principal component analysis (PCA) shows that the miRNA expression profiles in COVID-19 patients and healthy participants segregated into distinct clusters along principal component 1 and principal component 2 that explains 61% and 16% of the total variance, respectively (Fig. 1A). In order to identify miRNAs that were significantly differentially expressed between the two groups, volcano plot filtering was performed (Supplementary Fig. 1). The cut-off threshold for significantly differentially expressed miRNAs was a fold-change ≥10 and P < 0.05. A total of 8 miRNAs were shown to be differentially expressed between the COVID-19 patients and healthy participants. The expression of top 8 differentially expressed miRNAs are depicted in heat maps, in which the expression levels are assigned a colour value (Fig. 1B). The expression of miR-150-5p, miR-375, miR-122-5p, and miR-494-3p were significantly down-regulated in COVID-19 patients with moderate-severe disease (Fig. 2A). We also observed a significant up-regulation in the expression levels of miR-3197, miR-4690-5p, miR-1915-3p, and miR-3652 in all the above COVID-19 patients when compared to healthy participants (Fig. 2B). The above results from the extraction-free HTG EdgeSeq technology for the 8 circulating miRNAs were authenticated by performing RT-qPCR (Fig. 3A, B). We used miR-155-5p as a control because the circulatory levels of it in the COVID-19 patients was comparable to what was observed in healthy participants (data not shown). These results demonstrated a significant difference in the circulating miRNA profiles in COVID-19 patients compared to healthy participants.

### Pleiotropic effect of the changes in the miRNA expression profile in COVID-19 patients

**Figure 4.**
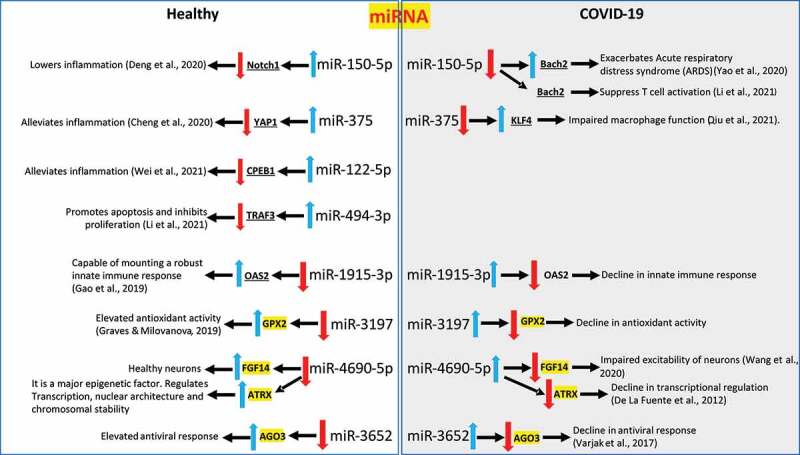
**Pleiotropic effect of changes in the expression profiles of the circulating miRNAs in COVID-19 patients compared to healthy individuals**. A schematic demonstrating the net effect of changes in circulating miRNA profiles in COVID-19 patients with moderate-severe disease when compared to healthy volunteers is presented. The direct published and confirmed targets of the miRNAs are ‘underlined’. The predicted targets of the miRNAs are highlighted in ‘*yellow*’ as there is no published data available as of this date. The red and blue arrows denote down-regulation or upregulation of miRNAs or the target genes, respectively.

Decrease in the expression of miR-150-5p, miR-375, miR-122-5p, and miR-494-3p in COVID-19 patients is a sign of inflammation and an impaired immunity [10,26–31] (Fig. 4). Increase in the expression miR-1915-3p, miR-3197, miR-4690-5p, and miR-3652 may result in a significant decline of transcriptional activity, innate immunity, and antioxidant activity [32–36] (Fig. 4). Luciferase assays were performed to confirm the ability of a select few cellular miRNAs to bind and physically interact with their targets and the data is provided in Supplementary Fig. 2. The overall result of the changes to miRNA expression profile observed in COVID-19 patients enhanced inflammation, cell death and tissue damage while lowering the immunity.

### miR-150-5p mimic inhibit SARS-CoV-2 infection of cells

**Figure 5.**
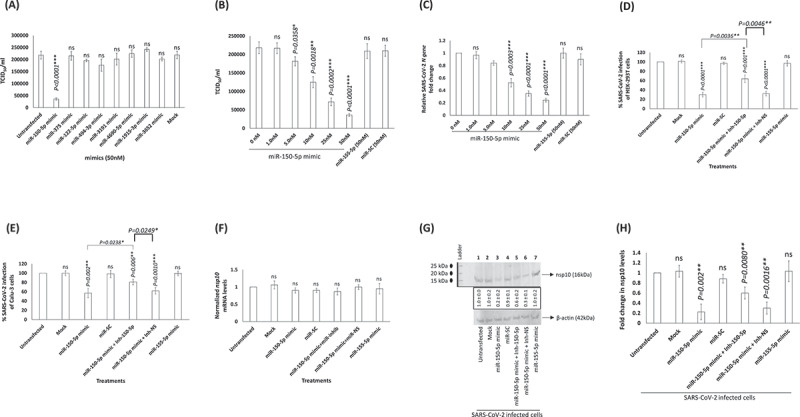
**miR-150-5p overexpression inhibits SARS-CoV-2 infection of cells. (A, B, C) Expression of miR-150-5p mimic significantly lowers SARS-CoV-2 infection**. (A) HEK-293 T cells were transiently transfected with 50 nM concentration of different miRNA mimics. At 24 h post transfection, these cells were infected with 1MOI of SARS-CoV-2. On 3^rd^ day PI, the supernatant was collected, and the virus infectivity monitored in Vero cells and presented as TCID_50._ (B) In another set of experiments, the effect of different concentrations of miR-150-5p mimic on SARS-CoV-2 infection was assessed as above and the data presented as TCID_50_. (C) The viral load in the culture supernatant of cells treated with different concentrations of mimics and subsequently infected with SARS-CoV-2 was monitored by qPCR (for *N* gene expression). The relative fold change in the expression of *N gene* in cells transfected with various mimics compared to the untransfected cells (0 nM) is presented. **Transfection of cells with Inh-150-5p opposes the effects of miR-150-5p mimic on SARS-CoV-2 infection of HEK-293 T** (D) **and Calu-3** (E) **cells**. Cells were either untransfected, mock transfected, transiently transfected with miR-150-5p mimic, scramble control (miR-SC), co-transfected with miR-150-5p mimic and Inh-150-5p, or co-transfected with miR-150-5p mimic and Inh-NS before infection with SARS-CoV-2. Data was plotted to represent the percentage of SARS-CoV-2 infection in transfected cells compared to untransfected cells. (F) RT-qPCR and (G) Western blotting analysis were performed for *nsp10* mRNA and nsp10 protein levels, respectively, in the above SARS-CoV-2 infected cells. The blot is representative of four different experiments. (H) The above Western blotting data representing the nsp10 protein expression levels under various treatment conditions are presented as fold change compared to nsp10 levels in SARS-CoV-2 infected but untransfected cells (average ± s.d. from three experiments). Bars represent average ± s.d of four (panels A – F, H) individual experiments. Student *t* test was performed to compare groups. Two-tailed *P* value of 0.05 or less was considered statistically significant. *P < 0.05; **P < 0.01; ***P < 0.001; ns-not significant.

We analysed the effects of the above 8 different miRNAs (Fig. 4) to alter *in vitro* SARS-CoV-2 infection. This was attempted by using specific miRNA mimics. The range of mimic doses tested in this study is comparable to those reported in the earlier studies [5,37]. The miRNA mimics from 10 nM to 50 nM did not seem to significantly kill cells (Supplementary Fig. 3). Of all the miRNA mimics tested, it was miRNA-150-5p mimic that could significantly alter SARS-CoV-2 infection of HEK-293 T cells (Fig. 5A). To evaluate the biological effects of miR-150-5p in target cells, we analysed the effects of Inh-150-5p on SARS-CoV-2 infection of HEK-293 T cells. The Inh-150-5p and Inh-NS up to 50 nM concentration was determined to be non-lethal to cells as monitored by LDH assay (Supplementary Fig. 4). To further authenticate the effect of miR-150-5p mimic on SARS-CoV-2, we analysed the effect of transfecting HEK-293 cells with the mimic and corresponding inhibitor on miR-150-5p expression. The expression of miR-150-5p in the cells transfected with miR-150-5p mimic was significantly increased compared with the cells transfected with miR-SC controls (Fig. 5). The mimic-induced miR-150-5p expression levels were significantly lowered following the treatment of Inh-150-5p in comparison with Inh-NS (Supplementary Fig. 5).

The titre of SARS-COV-2 in the culture supernatant collected 3 days PI from HEK-293 T cells transfected with 50 nM concentration of miR-150-5p mimic was significantly lower when compared to those cells that were untransfected, transfected with scrambled control, or miR-155-5p mimic (Fig. 5B). We observed a dose response inhibitory effect of miR-1505p mimic on SARS-CoV-2 infection of cells (Fig. 5B). These results were further confirmed by performing RT-qPCR by monitoring the expression of N gene using appropriate primer/probe combination (Fig. 5C). Incidentally, the effect of miR-150-5p mimic on SARS-CoV-2 infection of HEK-293 T cells could be significantly reversed by co-transfecting cells with 25 nM of Inh-150-5p (Fig. 5D). Co-transfection of cells with Inh-NS (non-specific inhibitor) did not alter the effects of miR-150-5p mimic (Fig. 5D). We observed identical inhibitory trend in Calu-3 cells (Fig. 5E). The inhibition observed in Calu-3 cells varies greatly when compared to HEK-293 T. The variation in cell types is an important factor here and while the trend is identical, the results are not. To corroborate the above effects of miR-mimics on SARS-CoV-2 and the actual *nsp10* mRNA levels, we performed RT-qPCR. We detected no change in endogenous *nsp10* mRNA levels (Fig. 5F). However, there was a significant decrease in the nsp10 protein levels in SARS-CoV-2 infected HEK-293 T cells transfected with miR-150-5p mimic (Fig. 5G; *lane 3*) compared to untransfected cells (Fig. 5G; *lane 1*) and those cells that were transfected with miR-155-5p mimic (Fig. 5G; *lane 7*). The effects of transfecting SARS-CoV-2 infected cells with miR-150-5p mimic could be significantly reversed by co-transfecting cells with Inh-150-5p (Fig. 5G; *lane 5*; Fig. 5H) compared to co-transfecting cells with miR-SC (Fig. 5G; *lane 4*; Fig. 5H). The results demonstrate the following: (i) specificity of the effects of miR-150-5p mimic on SARS-CoV-2 infection of cells; and (ii) the interactions between the miR-150-5p and the MREs located in the coding strand (CDS) of *nsp*10 repress translation than mRNA decay.

**Figure 6.**
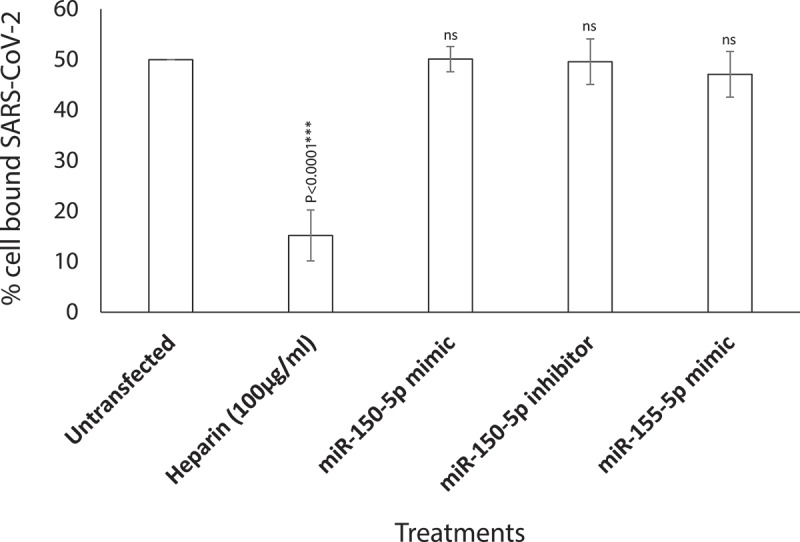
**miR-150-5p mimic inhibition of KSHV infection of cells is at a post-attachment stage of SARS-CoV-2 infection**. SARS-CoV-2 binding to HEK-293 T cells that were untransfected, transiently transfected with 50 nM of miR-150-5p mimic, 25 nM of Inh-150-5p, 50 nM of miR-155-5p mimic, or untransfected and treated with 100 µg/ml of heparin was monitored by performing binding assay. Data was plotted to represent the percentage of SARS-CoV-2 binding to target cells treated differently compared to the untransfected cells. Bars represent average e ± s.d of five individual experiments. Student *t* test was performed to compare different treatments with the untransfected cells. Two-tailed *P* value of 0.05 or less was considered statistically significant. ***P < 0.001; ns – not significant.

To ascertain the stage at which the miR-150-5p mimic altered SARS-CoV-2 infection, we performed a binding assay. The binding assay performed on HEK-293 T cells demonstrated that miR-150-5p mimic and the inhibitor did not block SARS-CoV-2 from binding the target cells (Fig. 6). Incubating SARS-CoV-2 with soluble heparin significantly blocked SARS-CoV-2 from binding cells (Fig. 6). Our results clearly implicate miR-150-5p to inhibit SARS-CoV-2 infection of cells at a step post binding to cells.

### miR-150-5p may potentially targets SARS-CoV-2 encoded nsp10 gene

**Figure 7.**
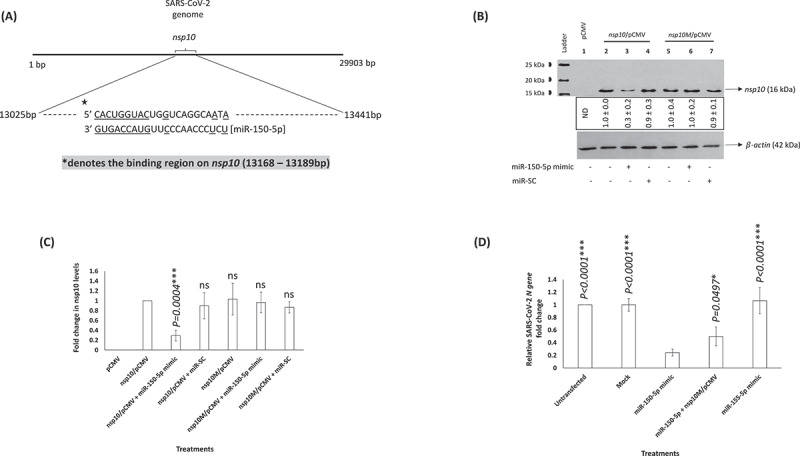
(A) **miR-150-5p putative binding sites**. A schematic showing the predicted putative binding sites of miR-150-5p in the coding strand of *nsp10* gene. The binding sites are underlined. (B) SARS-CoV-2 encoded *nsp10* is a potential target of miR-150-5p. HEK-293 T cells were either transfected with pCMV empty vector (*lane 1), nsp10*/pCMV (*lanes 2, 3, 4*), or *nsp10M*/pCMV (*lanes 5, 6, 7*). These cells were subsequently untransfected, transfected with 50 nM of miR-150-5p mimic, or miR-SC. At 24 h post transfection the cells were lysed, protein concentration determined, lysate resolved in a 15% SDS-PAGE gel, transferred on to a PVDF membrane and probed with appropriate antibodies as per standard protocols. (C) The above Western blotting data representing the nsp10 protein expression levels under various treatment conditions are presented as fold change compared to nsp10 levels in cells transfected with *nsp10*/pCMV (average ± s.d. from three experiments). Student *t* test was performed to compare different treatments with cells transfected with *nsp10*/pCMV. Two-tailed *P* value of 0.05 or less was considered statistically significant. ***P < 0.001; ns – not significant. (D) HEK-293 T cells were either untransfected, mock transfected, transiently transfected with 50 nM miR-150-5p mimic, miR-155-5p mimic, or co-transfected with miR-150-5p mimic and *nsp10M*/pCMV before infection with SARS-CoV-2. The viral load in the culture supernatant of cells treated with different concentrations of mimics and subsequently infected with SARS-CoV-2 was monitored by RT-qPCR (for *N* gene expression). The relative fold change in the expression of *N gene* representing the virus particles in different treatments compared to untransfected cells is presented. Student *t* test was performed to compare different treatments with cells transfected with miR-150-5p mimic. Two-tailed *P* value of 0.05 or less was considered statistically significant. ns – not significant.

*In vitro* studies demonstrated the ability of miR-150-5p mimic to lower SARS-CoV-2 infection of cells. The ability of the miR-150-5p mimic to lower virus infection could be as a result of directly targeting a viral gene or by an indirect effect via targeting a host cell gene expression. As a first step, we tried to map and determine if miR-150-5p could target any viral gene. Our analysis showed that *nsp10* was a potential target of miR-150-5p (Fig. 7A). The schematic reveals an extensive 3ʹ base-pairings in the miR-150-5p seed. To establish the direct effect of miR-150-5p on the expression of *nsp10* gene, we transiently transfected HEK-293 T cells with pCMV, *nsp10*/pCMV, *nsp10M*/pCMV. These cells were further transfected with miR-150-5p mimic or scramble control (miR-SC) prior to monitoring the expression of nsp10 by Western blotting. Our data demonstrate the ability of miR-150-5p mimic to specifically lower the expression of the nsp10 protein encoded by the wild type *nsp10* gene (Fig. 7B; *lane 3*; Fig. 7C). However, miR-150-5p mimic expression could not lower the levels of the nsp10 protein encoded by *nsp10 gene* lacking the wild-type MRE (Fig. 7B; *lane 6*; Fig. 7C). Taken together, SARS-CoV-2 encoded *nsp10* may be a novel target for the cellular miR-150-5p. Finally, we attempted to determine if expression of nsp10M could rescue SARS-CoV-2 infection in cells transfected with miR-150-5p mimic. Our results demonstrated endogenous expression of nsp10M could partially rescue SARS-CoV-2 infection in cells that were transfected with miR-150-5p mimic (Fig. 7D). The data suggests nsp10M protein to structurally mimic wild-type nsp10 protein and that the miR-150-5p may target more than *nsp10* mRNA in SARS-CoV-2 infected cells. Overall, our study concludes an important role for miR-150-5p in modulating nsp10 expression and thereby alter SARS-CoV-2 infection.

### Discussion

The original intent of this project was to probe for changes in the circulatory miRNA profile in COVID-19 patients with moderate-severe disease. Accordingly, we performed a direct comparison of the miRNA expression profile between COVID-19 positive patients with moderate-severe disease and healthy participants. This was conducted using the EdgeSeq miRNA whole-transcriptome assay to provide novel ideas to unravel the pathways underlying the biology of COVID-19 in patients. A recent study estimated a total of 2,300 mature miRNAs to be present in human body [38]. Of these, more than 50% have been annotated in miRBase. The EdgeSeq miRNA whole transcriptome assay allowed us to screen for approximately 2083 miRNAs in the human plasma that accounts for approximately 91% of the miRNome.

In a recently concluded study, miRNA profile in COVID-19 patients was reported [17]. The study by Li et al., differed from our study as follows: (i) the patients showed mild/moderate illness; (ii) all the patients were on antivirals that were not listed; and (iii) identified miR-618 as a promising therapeutic and diagnostic target to treat COVID-19. This miRNA has a crucial role to play in tumour biology [39]. In another recently published study by Mi et al., it was demonstrated that the SARS-CoV-2-induced overexpression of miR-4485 could directly suppress osteogenic differentiation and fracture healing [40]. This study differed from our study in that the orthopaedic patients selected for the study were either uninfected or SARS-CoV-2 infected once upon a time and were negative for viral RNA at the time of admission to this study. Current study such as this that profile miRNome have their own share of challenges that includes specimen collection, storage of specimens, handling, and data analysis; all of which may equally contribute to contradictory results [41]. Of these, we consider specimen collection and storage to be vital determinants as it was a challenge during the worst phase of the pandemic. One other challenge we had was in classifying the COVID-19 patients as with ‘moderate-severe disease’ instead of two separate categories (moderate vs severe disease). This is because of the following reasons: (i) the classification was purely based on the symptoms noticed at the time of admission and when the blood for plasma was drawn; (ii) at that time, all the patients were confirmed SARS-CoV-2 by PCR, had moderate-severe pneumonia as assessed by radiographic imaging and oxygen requirements; (iii) all the participants were requiring supplemental oxygen at the time of the blood sampling. However, none of them required mechanical ventilation (meaning critical stage). Therefore, it was difficult for us to sort the patients further into moderate and severe disease group at such an earlier time.

Herein, we report three crucial findings. The first pertains to significant changes in the miRNA profile of COVID-19 patients with moderate-severe disease compared to healthy people. (Figs. 1–3). The second finding is on the ability of miR-150-5p mimic to inhibit *in vitro* SARS-CoV-2 infection of cells (Fig. 5). Finally, we provide preliminary evidence to demonstrate the ability of miR-150-5p to alter SARS-CoV-2 infection of cells (Fig. 5D-F) via regulating the expression of virus encoded *nsp10* (Fig. 7). It is important to mention here that there was no significant link observed between the admission 25-hydroxy-vitamin D, CRP, and ALC levels and the disease outcome (Table 1). A recent report concluded that the inflammatory markers are not good prognostic biomarkers [42].

The miRNome identified in this study for healthy controls compare well with those reported in other studies. For example, miR-150-5p is elevated in healthy people while significantly lower in critically ill COVID-19 patients [43]. miR-375 is highly expressed in pancreas and is required for normal glucose homoeostasis in healthy people [44]. miR-122-5p is actually lowered in COVID-19 patients compared to healthy participants. miR-122 has been predicted based on the minimum free energy of formation (MFE) values to directly bind to SARS-CoV-2 RNA genome [45]. The authors are not sure of the effects of such interactions between miR-122 and SARS-CoV-2 genome. They predict that such an interaction may also act as a suppressor of SARS-CoV-2. This makes sense as higher levels of miR-122 in healthy people can successfully thwart SARS-CoV-2 infections. Finally, miR-494 expression is elevated in healthy volunteers compared to COVID-19 patients. Recent study predicted miR-494-3p to have a role of providing resistance to SARS-CoV-2 infection [46]. Once again, this fits in our model as healthy people with elevated levels of circulating miR-494 are resistant to SARS-CoV-2 infections. In a way, these evidence not only authenticate our results but also validate the accuracy of the EdgeSeq system used in this study. At this juncture, we would like to point to the fact that miR-155-5p was not significantly altered in COVID-19 patients with moderate-severe disease and hence we used it as a control in some of our experiments (Figs. 3, 5, 6, 7D). On the contrary, another study reported miR-155 to be elevated in critically ill mechanically ventilated COVID-19 patients. This discrepancy in the data could be due to differences in the patient groups. None of the patients in our study were on mechanical ventilation.

Viruses are known to hijack host gene expression and modulate fundamental cellular processes to establish infection and pathogenesis [47]. Several studies have demonstrated crucial roles for host cell miRNAs during viral infections [5,48]. A miRNA can regulate multiple targets including that of host and viral genes [5,49]. In order to understand the biological and cellular effects of the eight different miRNAs (Figs. 1–3) that were significantly altered in COVID-19 patients with moderate-severe disease, we used various bioinformatic tools as well as published reports. The three different tools used in this study were DIANA, miRmap, and miRDB [50–52]. Our approach was (i) to determine the putative target genes using the above tools; and (ii) to review published literatures and authenticate the putative targets for the specific miRNAs. By this approach, we successfully built a model describing the wholistic effects of the cellular miRNAs on COVID-19 pathology (Fig. 4). The overall result of the changes to miRNA expression profile in COVID-19 patients were enhanced inflammation, cell death and tissue damage; accompanied by a decrease in immunity.

To rule out any plausible direct effect of these circulating miRNAs (Fig. 4) on SARS-CoV-2 infection of cells, we performed infection assays using HEK-293 T cells. HEK-293 T cells supported only modest viral replication as reported earlier [53]; however, that was sufficient for this study as we were monitoring relative effects of various mimics and inhibitors compared to untransfected controls. Of all the miRNA mimics tested, miR-150-5p mimic was the only one to significantly inhibit *in vitro* infection of SARS-CoV-2 in HEK-293 T cells (Fig. 5). It was concluded that the effects of miR-150-5p mimic and inhibitor on SARS-CoV-2 infection was at a post attachment stage of internalization as they did not adversely affect virus binding to cells (Fig. 6). Identical results were obtained in a pilot study conducted using Calu-3 cells (Supplementary Fig. 5). However, the inhibitory effects of miR-150-5p mimic on SARS-CoV-2 infection of Calu-3 cells was not as pronounced as in HEK-293 T cells. This could be as a result of variations in the cell-types.

If miR-150-5p mimic could inhibit *in vitro* SARS-CoV-2 infection, we hypothesized miR-150-5p to directly interact and regulate a viral transcript. We identified a putative MRE with the potential to regulate *nsp10* gene. This is a novel MRE as it was not found in the traditional 3ʹ-UTR regions but found as an atypical MRE in the CDS of *nsp10* (13,168–13189bp) (Fig. 7A). In our study, endogenous expression of miR-150-5p mimic could not lower *nsp10* transcription (Fig. 5E) but could significantly inhibit nsp10 protein expression in HEK-293 T cells (Fig. 5F). Our working model considers that the cellular miR-150-5p binds the MRE that is contained within the CDS sequence of *nsp10*. The CDS-targeted miRNAs require extensive base pairings in the 3ʹ side rather than the 5ʹ seed to repress translation by inducing transient ribosome stalling instead of mRNA destabilization [54–56]. By this way, we hypothesize miR-150-5p to exercise a more precise regulation of nsp10 without actually hampering the translation of downstream mRNA segments.

The specificity of miR-150-5p interactions with *nsp10* was confirmed by using *nsp10M*/pCMV that lacked a functional MRE (Fig. 7B, C). Also, expression of nsp10M could only partially rescue SARS-CoV-2 infection in cells transfected with miR-150-5p mimic. The data suggest that the miR-150-5p may target more than *nsp10* mRNA in SARS-CoV-2 infected cells; including perhaps, multiple cellular (Fig. 4) /viral target genes. The rescue experiment with *nsp10M* is an important result but the caveats associated with transfecting plasmid and miRNA mimics together are understandable in addition to the expression of virus particle, and the temporal dynamics of endogenous *nsp10* and associated viral proteins. SARS-CoV-2 nsp10 acts as a co-factor to the 2′-O-RNA methyltransferase (MTase) function of nsp16 [57]. Methylation of the 5′-end of virally encoded mRNAs by the nsp10/nsp16 dimer [58] mimics cellular mRNAs and thereby protect the virus from host innate immune response [59]. A recent study determined the ability of nsp10 to activate the 3ʹ to 5ʹ exoribonuclease (ExoN) activity of nsp14 aiding in immune evasion [22]. Therefore, nsp10 plays a crucial role in SARS-CoV-2 pathogenesis. Interestingly, miR-150 was predicted by earlier computational studies to specifically bind SARS-CoV-2 genome using different prediction algorithms [60]. The prediction-based studies concluded that miRNA interactions with the SARS-CoV-2 genome may have antiviral activity or increase stability and thereby promote viral replication. These results may have direct implications on not only COVID-19 but also on the viral replication.

Based on the results, we predict SARS-CoV-2 infection to alter expression of miR-150-5p directly or indirectly in host cells. Virus may interfere with miRNA biogenesis by directly competing with cellular pre-miRNAs or indirectly influencing miRNA expression due to host immune responses to extraneous agent. In summary, our study on plasma miRNAs describes a unique and reliable pattern of noninvasive miRNA-based biomarkers of COVID-19 patients. The work also helped us establish a pathophysiological link between a decrease in the miR-150-5p levels in COVID-19 patients to a possible increase in SARS-CoV-2 infection. Preliminary evidence supports the ability of miR-150-5p to possibly interact with its viral target (*nsp10*) to lower infection of cells. Further investigations on the miR-150-5p: *nsp10* interactions will help us get closer to better appreciate the biology of COVID-19.

## Supplementary Material

Supplemental Material
