# Supplementary material for: Cellular miR-150-5p may have a crucial role to play in the biology of SARS-CoV-2 infection by regulating nsp10 gene
Source: RNA Biol. 2021 Dec 14;19(1):1–11. doi: 10.1080/15476286.2021.2010959 (PMC8786335; doi:10.1080/15476286.2021.2010959)
Supplement: Supplemental Material [file KRNB_A_2010959_SM9189.zip › supplementary/downloadFromZipFile.pdf]

Supplementary Table S1: Forward primers to amplify miRNAs.

S.No.	miRNAs	Forward primer
**1**	miR-150-5p	5’-TCTCCCAACCCTTGTACCAGTG-3’
**2**	miR-375	5’-GCGACGAGCCCCTCGCACAAACC-3’
**3**	miR-122-5p	5’-TGGAGTGTGACAATGGTGTTTG-3’
**4**	miR-494-3p	5’-AGGTTGTCCGTGTTGTCTTCTCT-3’
**5**	miR-3197	5’-GGAGGCGCAGGCTCGGAAAGGCG-3’
**6**	miR-4690-5p	5’-GAGCAGGCGAGGCTGGGCTGAA-3’
**7**	miR-1915-3p	5’-ACCTTGCCTTGCTGCCCGGGCC-3’
**8**	miR-3652	5’-CGGCTGGAGGTGTGAGGA-3’
**9**	miR-155-5p	5-TTAATGCTAATCGTGATAGGGGTT-‘3

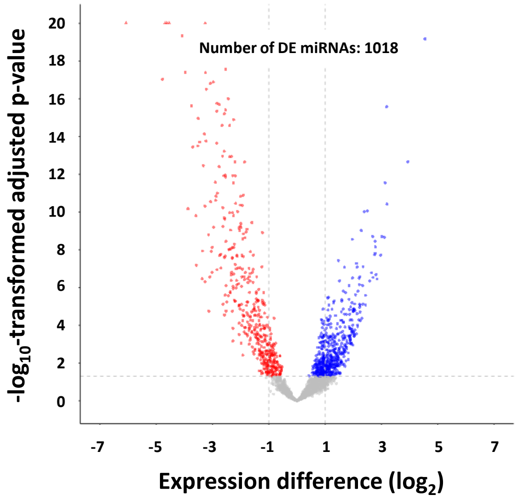

Supplementary Figure 1: Volcano plot for differentially expressed miRNAs, showing distribution of significance [-log10(p-value)] vs. fold change [log2(fold change)] for all genes. The red and blue dots represent miRNA expression in COVID-19 patients and healthy participants, respectively.

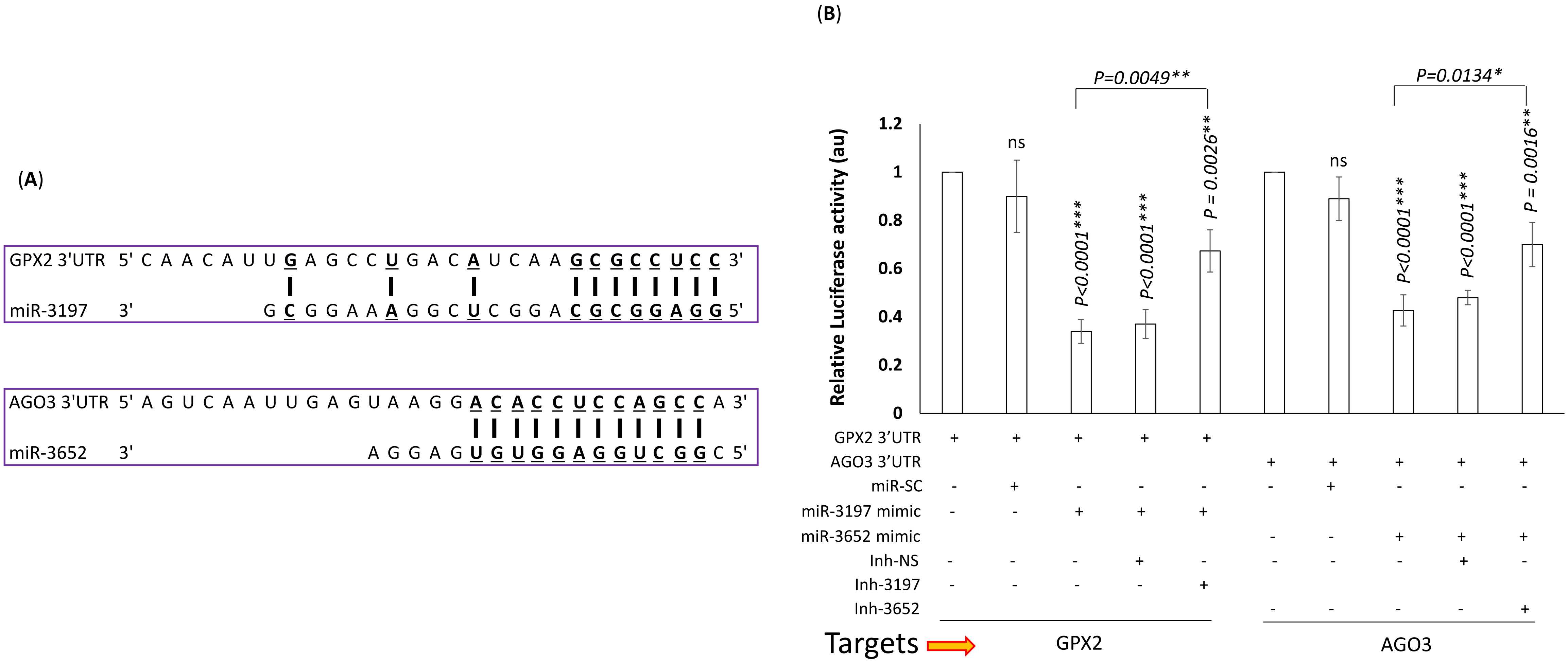

Supplementary Figure 2: (A) The prediction software (DIANA: http://diana.imis.athena-innovation.gr/DianaTools/index.php?r=site/index) suggested the targets (GPX2 and AGO3) have a binding site for miR-3197 and miR-3652, respectively, in its 3′UTR. **(B)** Dual luciferase reporter assay was performed to demonstrate the interactions between miRNAs (miR-3197 and miR-3652) and their respective targets (GPX2 and AGO3) in HEK-293T cells. The *x-axis* indicates the different treatments while the *y-axis* denotes the relative luciferase activity. Bars represent average±s.d. of three individual experiments. One-way analysis of variance (ANOVA) was performed using IBM SPSS v26 (Cary, NC) to determine significant differences between the different treatment and control groups, followed by Tukey HSD post-hoc test for multiple comparisons. The level of statistical significance is indicated with respective values; ns – not significant.

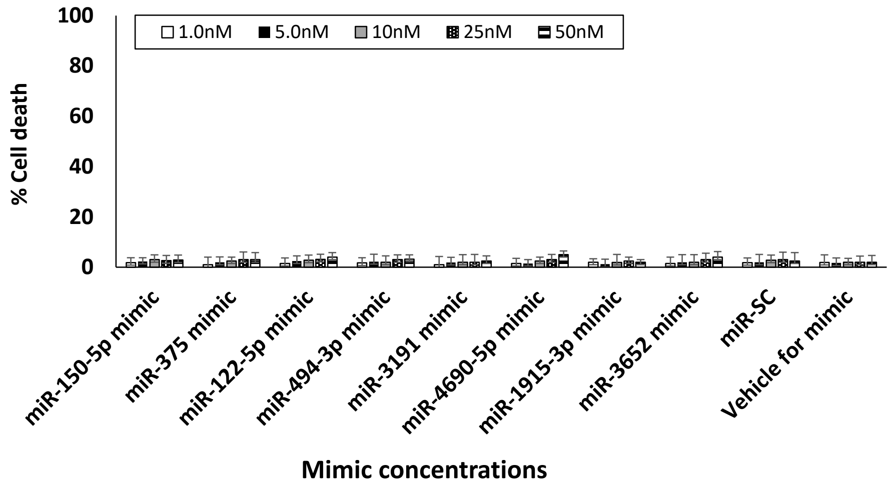

Supplementary Figure 3

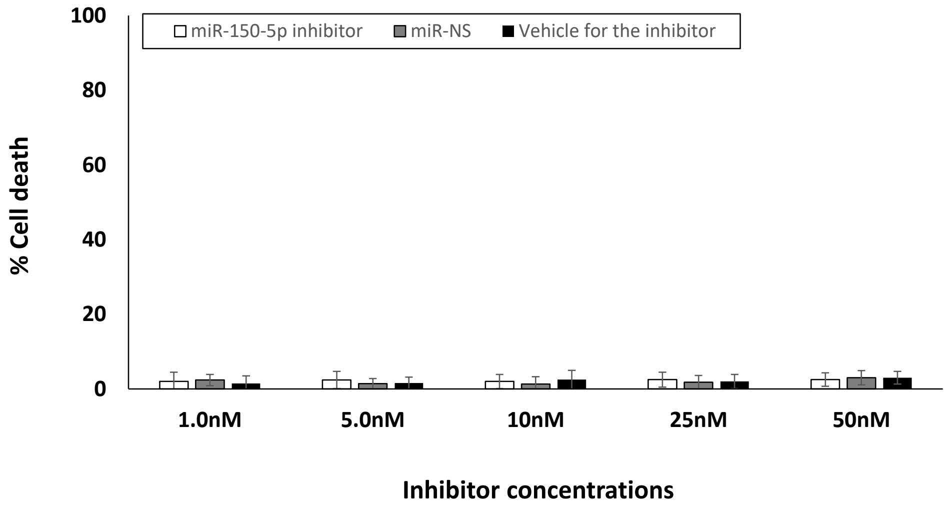

Supplementary Figure 4

Supplementary Figure 3: To determine the cytotoxic effect of the various miRNA mimics and miR-SC, cells were transfected with different concentrations of miRNA mimics. At 24h post transfection, lactate dehydrogenase release as an indicator of percentage of cell death was monitored for various mimics when compared to untransfected cells. Bars represent average e± s.d of five individual experiments.

Supplementary Figure 4: To determine the cytotoxic effect of the various concentrations of miR-150-5p inhibitor and miR-NS, cells were transfected with different concentrations of miR-150-5p inhibitors. At 24h post transfection, lactate dehydrogenase release as an indicator of percentage of cell death was monitored for miR-150-5p inhibitors when compared to untransfected cells. Bars represent average e± s.d of five individual experiments.

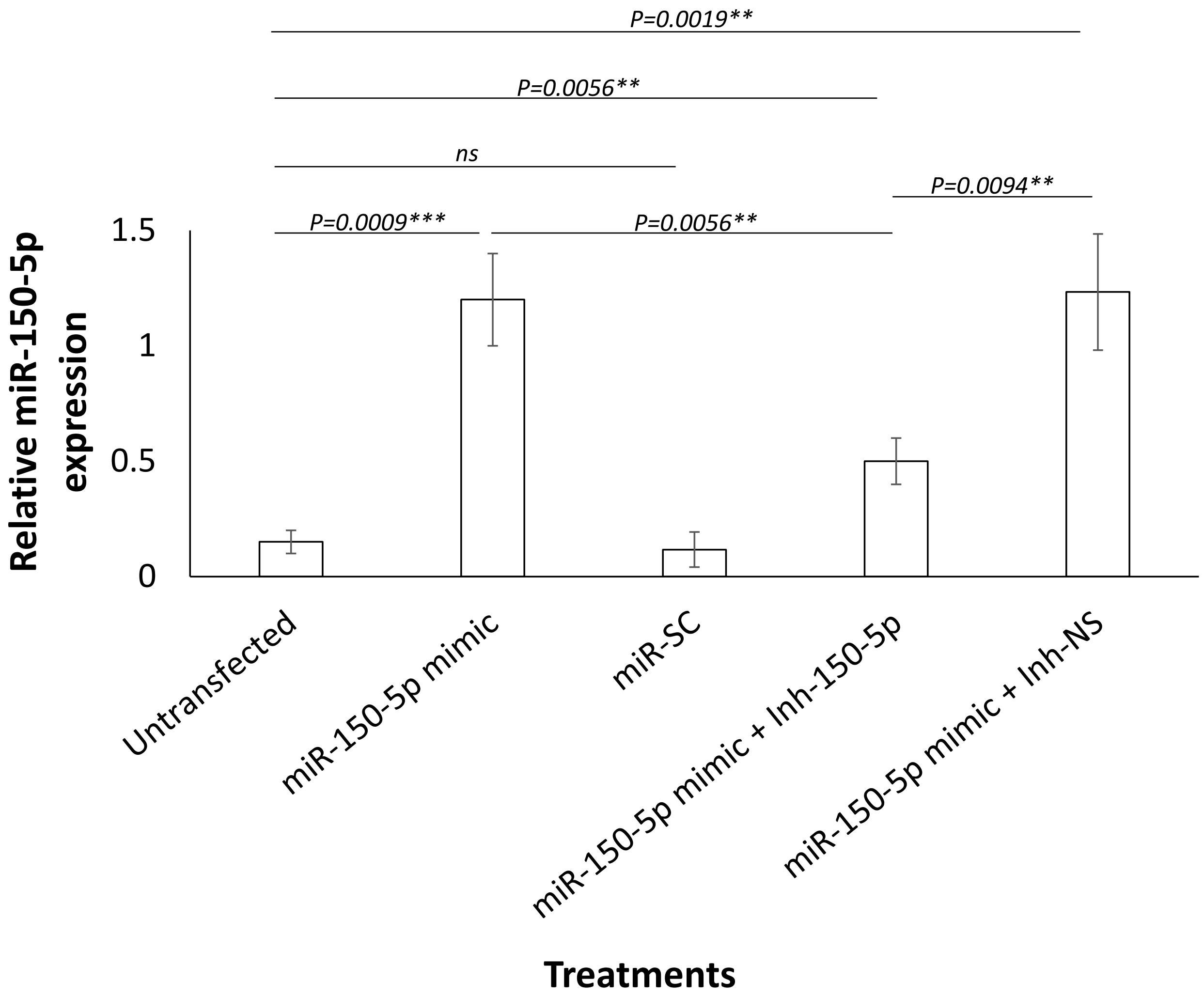

Supplementary Figure 5: miRNA expression and transfection efficiency in CRC cells. Expression levels of miR-150-5p in HEK-293 cells transfected with various oligos was detected at 24 h post-transfection by RT-qPCR. Bars represent average e± s.d of three individual experiments. . Student t test was performed to compare groups. Two-tailed *P* value of 0.05 or less was considered statistically significant. The level of statistical significance is indicated with respective values; ns – not significant.
